# Genetic Incorporation of Dansylalanine in Human Ferroportin to Probe the Alternating Access Mechanism of Iron Transport

**DOI:** 10.3390/ijms241511919

**Published:** 2023-07-25

**Authors:** Matteo Amadei, Antonella Niro, Maria Rosaria Fullone, Rossella Miele, Fabio Polticelli, Giovanni Musci, Maria Carmela Bonaccorsi di Patti

**Affiliations:** 1 Department of Biochemical Sciences ‘A. Rossi Fanelli’, Sapienza University of Rome, 00185 Rome, Italy; matteo.amadei@uniroma1.it (M.A.); mariarosaria.fullone@uniroma1.it (M.R.F.); rossella.miele@uniroma1.it (R.M.); 2 Department of Biosciences and Territory, University of Molise, 86090 Pesche, Italy; a.niro2@studenti.unimol.it (A.N.); giovanni.musci@unimol.it (G.M.); 3 Department of Biology, University Roma Tre, 00146 Rome, Italy; fabio.polticelli@uniroma3.it

**Keywords:** ferroportin, iron, MFS, genetic code expansion, dansylalanine, fluorescence spectroscopy, Pichia pastoris

## Abstract

Ferroportin (Fpn), a member of the major facilitator superfamily (MFS) of transporters, is the only known iron exporter found in mammals and plays a crucial role in regulating cellular and systemic iron levels. MFSs take on different conformational states during the transport cycle: inward open, occluded, and outward open. However, the precise molecular mechanism of iron translocation by Fpn remains unclear, with conflicting data proposing different models. In this work, *amber* codon suppression was employed to introduce dansylalanine (DA), an environment-sensitive fluorescent amino acid, into specific positions of human Fpn (V46, Y54, V161, Y331) predicted to undergo major conformational changes during metal translocation. The results obtained indicate that different mutants exhibit distinct fluorescence spectra depending on the position of the fluorophore within the Fpn structure, suggesting that different local environments can be probed. Cobalt titration experiments revealed fluorescence quenching and blue-shifts of λ_max_ in Y54DA, V161DA, and Y331DA, while V46DA exhibited increased fluorescence and blue-shift of λ_max_. These observations suggest metal-induced conformational transitions, interpreted in terms of shifts from an outward-open to an occluded conformation. Our study highlights the potential of genetically incorporating DA into Fpn, enabling the investigation of conformational changes using fluorescence spectroscopy. This approach holds great promise for the study of the alternating access mechanism of Fpn and advancing our understanding of the molecular basis of iron transport.

## 1. Introduction

Safe trafficking of iron across the cell membrane is a delicate process that requires specific protein carriers. Ferroportin (SLC40A1, Fpn) is the only known cellular iron export membrane protein identified so far in mammals and it is essential for physiological regulation of cellular and systemic iron levels [1]. Missense mutations of Fpn cause type 4 hereditary hemochromatosis (also known as ‘ferroportin disease’), a dominant form of hemochromatosis with parenchymal and/or reticulo-endothelial iron overload [2,3]. The recently reported cryoEM structure of human Fpn in complex with hepcidin has finally provided a framework for a clearer understanding of metal binding and has allowed us to propose a model of how the peptide regulates the transporter [4]. Experimental data have corroborated previous in silico predictions and confirm that Fpn belongs to the major facilitator superfamily (MFS) of secondary transporters [4,5,6,7,8]. Proteins belonging to this widespread group typically display a 12 TM organization (the ‘MFS fold’) and are believed to act with an alternating access mechanism that involves extensive conformational changes to translocate their substrate across the membrane. These include at least three different conformations, namely inward-open, occluded, and outward-open. An open question regards the molecular mechanism of iron translocation by Fpn; in fact, conflicting data have been reported suggesting that Fpn is a uniporter [9], a proton-coupled symporter [10], or a proton-coupled antiporter [11,12].

The conformational states of membrane proteins can be probed directly using fluorescence-based techniques. Analysis of the fluorescence of naturally occurring and/or engineered tryptophan residues has been widely employed. Additionally, chemical reactions that target cysteine thiol groups have been used to covalently attach environment-sensitive probes to proteins. However, these approaches can be challenging because they require the generation of a tryptophan- or cysteine-free background to achieve single-site labeling. Such limitations can be overcome by site-specific introduction of unnatural amino acids with unique functional groups that are not found in native proteins.

In this work the *amber* codon suppression strategy has been used to achieve site-specific incorporation of non-canonical amino acids into Fpn. *Amber* codon suppression relies on the ability of an engineered orthogonal aminoacyl-tRNA synthetase (aaRS) to charge its cognate tRNA with an unnatural amino acid and suppress a TAG *amber* stop codon placed within a coding sequence. This approach has been used to introduce genetically encoded unnatural amino acids to label target proteins selectively and site-specifically with different probes [13,14]. We show here that the fluorescent amino acid dansylalanine (DA) can be successfully introduced into human Fpn using this strategy. Fluorescence spectroscopy has been employed to investigate the potential of this approach for the study of the conformational changes in Fpn predicted to take place during the transport of iron.

## 2. Results and Discussion

### 2.1. Selection of Reporter Residues and Incorporation of Dansylalanine (DA) into Fpn

**Figure 1:**
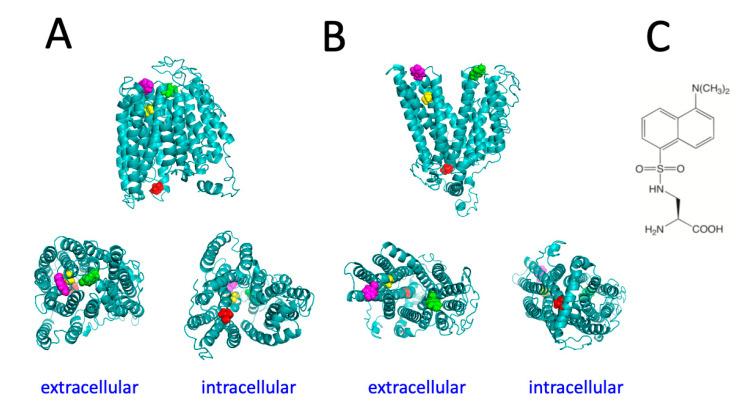
Structural models of the inward-facing conformation (**A**) and outward-facing conformation (**B**) of human Fpn. Residues V46 (yellow), Y54 (magenta), V161 (red), and Y331 (green) are shown in spacefill representation. (**C**) Structure of dansylalanine.

To identify regions of the protein predicted to undergo major conformational changes during the transport cycle, the structural models of human Fpn in different conformations [15] were employed, as this work was started before experimentally determined structures of Fpn became available. This preliminary analysis was performed with the purpose of selecting appropriate positions to place the unnatural amino acid DA. Of note, the recently solved structure of human Fpn turned out to be fully consistent with our in silico model, thus validating the selection of specific residues to be mutated for the present study. On the extracellular side of the protein, in the inward-open conformation, residue Y331, located on TM7, is close to Y54 that is found in the loop connecting TM1 and TM2 (Figure 1). When Fpn switches to the outward-open conformation these two residues are predicted to move away from each other and residue Y54 is predicted to substantially increase its solvent exposure (Figure 1). Therefore, positions 54 and 331 appeared to be suitable to act as sensors of the conformational transitions of Fpn. A sequence of three consecutive valines is found on the intracellular side of the protein in the loop connecting TM4 and TM5. Deletion of one of these valine residues was one of the first loss-of-function Fpn mutations identified, giving rise to hemochromatosis type 4A [16]. We therefore chose to mutate the central valine in the sequence, namely V161. Another valine, residue V46, was also chosen because it is located within TM1 in the channel through which iron is translocated. Changes in solvent accessibility between inward-open and outward-open conformations of Fpn are predicted to lead to decreased exposure of both these valine residues.

Fpn V46*amber*, Y54*amber*, V161*amber,* and Y331*amber* mutants were produced in order to exploit *amber* codon suppression in the yeast host *P. pastoris* to achieve the incorporation of dansylalanine (DA) into Fpn. The dansyl group of DA is an environment-sensitive fluorophore and this non-canonical amino acid has been used for the fluorescence spectroscopy analysis of protein unfolding [17]. Mutations Y54F and Y331F were shown to have no impact on the iron transport ability of Fpn [7], suggesting that amino acid changes in these positions are well-tolerated by the protein.

**Figure 2:**
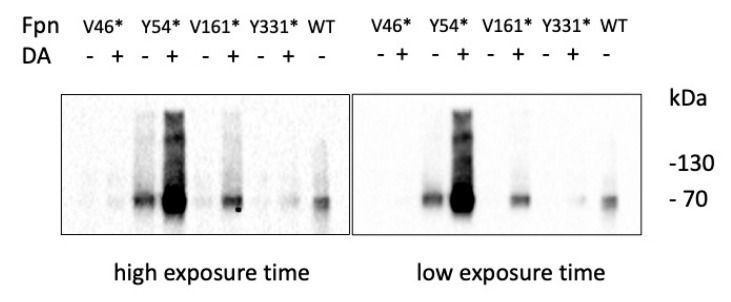
Expression of Fpn *amber* mutants. Western blot analysis of Fpn mutants ± DA.

*P. pastoris* strains expressing the tRNA/aaRS pair specific for DA and the Fpn mutants under control of methanol-inducible promoters were grown for 96 h in the presence of methanol with or without the unnatural amino acid. Western blot analysis evidenced the presence of full-length Fpn (ca. 62 kDa), indicating that suppression of the *amber* stop codon is effective and appears to be quite specific since much lower expression is found in cells grown in the absence of the unnatural amino acid (Figure 2). Expression levels varied, with Y54*amber* and V161*amber* showing significantly higher expression compared to V46*amber* and Y331*amber*.

**Figure 3:**
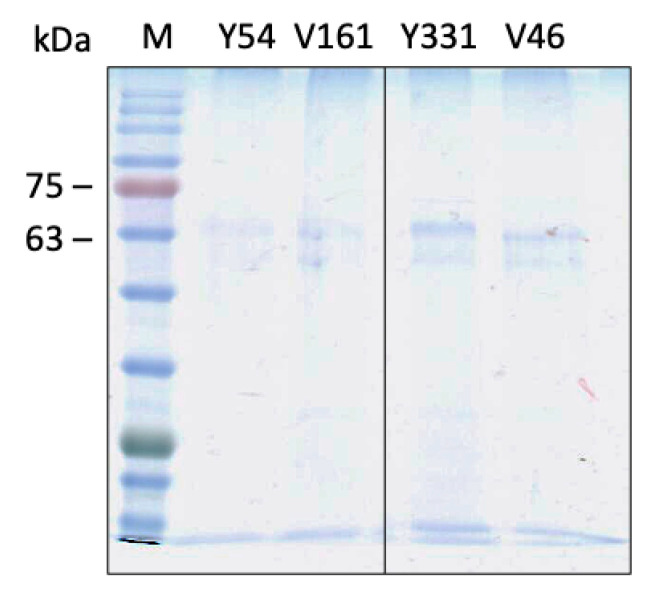
SDS-PAGE analysis of purified Fpn *amber* mutants.

Single-step purification of Fpn from membrane extracts was performed by immunoaffinity anti-FLAG chromatography. The purity of the protein was assessed by SDS-PAGE and was similar for all mutants (Figure 3). Yields of Fpn DA mutants were ca. 0.2–0.3 mg per liter of culture, somewhat lower than those obtained for wild type Fpn (ca. 0.5 mg per liter of culture). This result is in line with the consistently lower expression levels reported for other proteins incorporating genetically encoded unnatural amino acids compared to the wild type counterparts [17,18].

### 2.2. Fluorescence Spectroscopy of Fpn DA

Fluorescence spectroscopy analysis of Fpn was performed in order to evaluate the incorporation of the fluorescent probe. The dansylalanine fluorophore is maximally excited at 340 nm and emits at about 550–570 nm in aqueous solution [17]. The emission peak is significantly blue shifted when the dansyl group is in a hydrophobic milieu. Fluorescence of DA originates from an intramolecular charge transfer excited state in which electron density is transferred from the NMe_2_ group to the electron-accepting sulfonyl substituent of dansyl. Solvation and hydrogen bonding (together with the presence of nearby charged residues) affect emission maximum and quantum yield, making the dansyl group useful to probe the polarity and dynamics of the local environment in proteins [19].

**Figure 4:**
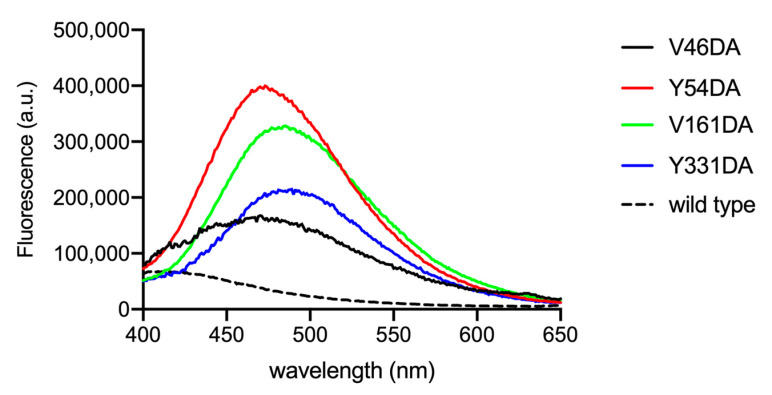
Fluorescence spectra of Fpn V46DA, Y54DA, V161DA, and Y331DA. Emission spectra were recorded in MOPS 25 mM pH 7.0, NaCl 150 mM, DDM 0.01%. Excitation was at 340 nm; the spectra are normalized on protein content determined by BCA. The spectrum of non-dansylated Fpn wild type is also shown.

The emission spectra of purified Fpn V46DA, Y54DA, V161DA, and Y331DA show a significantly blue-shifted peak with different maxima depending on the local position of the fluorophore (Figure 4). Human Fpn wild type (non-dansylated) showed a weak emission close to 400 nm, possibly due to the 10 tryptophan residues present in the protein (Figure 4, dashed line). This result confirms that the emission band in the 470–490 nm region can be attributed to the genetically incorporated non-canonical amino acid DA.

Among the mutants, V46DA appears to be the variant with the most hydrophobic environment surrounding dansylalanine (λ_max_ 470 nm), followed by Y54DA (λ_max_ 473 nm), V161DA (λ_max_ 485 nm), and Y331DA (λ_max_ 489 nm) with a 19 nm shift between V46DA and Y331DA. Based on the spectral properties of the mutants and the position of dansylalanine in the protein structure, interpretation of the resting state of Fpn is not clear-cut. A conformation more closely resembling the outward-open state could be in line with the observed λ_max_ values which indicate that positions 161 and 331 (which are found on opposite sides of the membrane bilayer) are in the most polar environment. This assumption is supported by the analysis of two recently reported outward-open structures of human Fpn solved by cryoEM (PDB 6W4S [4] and 8C03 [20]) which indicates that accessible surface area (ASA) is highest for Y331 (53–47 Å^2^), followed by V161 (42–31 Å^2^) and confirms that V46 is buried (ASA 10–9 Å^2^). The calculated solvent-accessible area for Y54 yields two significantly different values (ASA 61 Å^2^ for 6W4S or 27 Å^2^ for 8C03) possibly due to slight changes in the position of TM2 and of the loop connecting TM3 and TM4 in the two structures, suggesting a certain degree of flexibility in this region. It should be pointed out that the two structures differ for the absence (6W4S) or presence (8C03) of the inhibitor vamifeport in the central cavity of Fpn. The λ_max_ value for the Y54DA protein is consistent with a less polar environment compared to Y331DA and V161DA. Whether this is correlated to a more buried location of Y54 or to local higher hydrophobicity due to other factors, such as a lack of neighboring hydrogen bonding or charged residues, remains to be established.

### 2.3. Cobalt Titration

To evaluate whether dansylalanine can be useful to probe the conformational changes that Fpn undergoes during metal translocation, all DA mutants (1 µM) were titrated with CoCl_2_ (0–52 µM), which is more stable than Fe^2+^ and has been routinely employed as Fe substitute.

**Figure 5:**
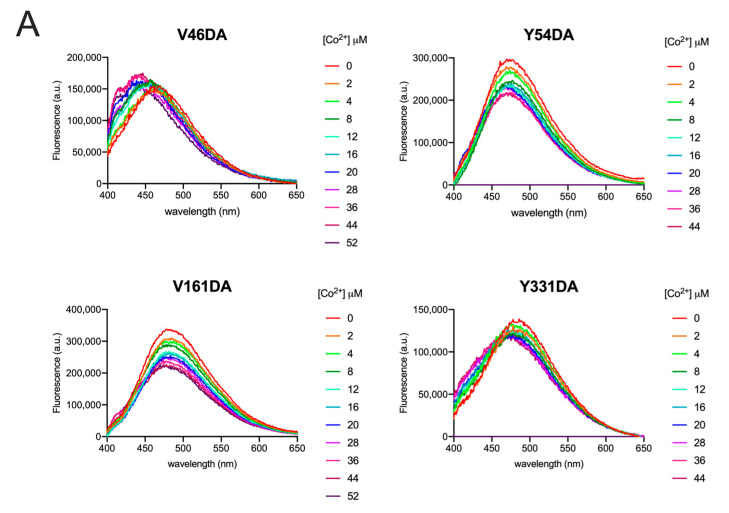

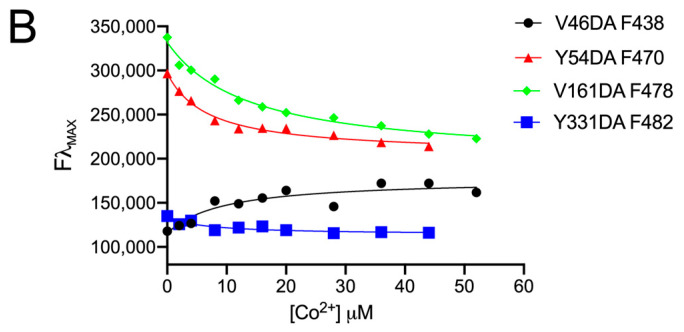
Fluorescence spectra of Fpn V46DA, Y54DA, V161DA, and Y331DA titrated with cobalt. (**A**) Emission spectra of Fpn 1 µM after addition of the indicated concentration of CoCl_2_ were recorded in MOPS 25 mM pH 7.0, NaCl 150 mM, DDM 0.01%. Excitation was at 340 nm. The spectrum of Fpn wild type was subtracted as baseline. (**B**) Fluorescence changes at λ_max_ for each Fpn DA mutant. Data were fitted to a one-site binding model.

Fluorescence spectra (Figure 5A) were collected immediately after metal addition, and fluorescence intensity values at λ_max_ for each mutant were then plotted as a function of the metal concentration (Figure 5B). To better evaluate changes in the DA probe, spectra obtained for the wild type (non-dansylated) Fpn (1 µM) at 340 nm excitation were subtracted from all spectra of the DA mutants.

All data were fitted to a one-site binding model to obtain K_D_ values (Table 1). Although two cobalt-binding sites are found in the 3D structure of human Fpn [4], fitting the data to a two-site binding model was very poor, yielding ambiguous results. Thus, it may be hypothesized that the affinity of the two sites for cobalt is similar, thus making a one-site binding model appropriate.

All mutants exhibited a similar affinity for Co^2+^ (K_D_ 6–14 µM). The K_D_ for Fpn wild type was determined in the same conditions except that excitation was at 295 nm and the protein was 0.1 µM to avoid saturation of the fluorescence signal (Figure S1). The K_D_ values are in line with K_M_ values in the low µM range reported for the transport activity of Fpn reconstituted in liposomes and assayed with calcein [10,20].

Following the addition of cobalt, Y54DA, V161DA, and Y331DA exhibited a similar trend with a significant quenching of fluorescence and limited blue shifts of λ_max_ (5–10 nm). V46DA, instead, showed an opposite behavior: increasing DA fluorescence during metal titration and blue shifting its λ_max_ by about 15 nm.

**Figure 6:**
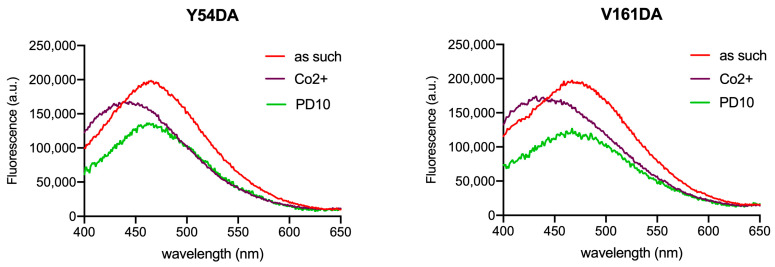
Fluorescence spectra of Fpn Y54DA and V161DA after the removal of CoCl_2_. Emission spectra of Fpn 1 µM as such, after titration with cobalt (36/28 µM), and after gel-filtration (PD10) were recorded in MOPS 25 mM pH 7.0, NaCl 150 mM, DDM 0.01%. The PD10 spectrum is arbitrarily scaled to evidence the λ_max_ position. Excitation was at 340 nm.

Size exclusion chromatography (SEC) on a PD10 desalting column was performed to remove CoCl_2_ from samples in order to check the reversibility of the observed changes. Changes affecting λ_max_ appeared to be fully reversible, as demonstrated by the spectra measured after the removal of cobalt by SEC. Figure 6 shows the spectra for Fpn Y54DA and V161DA; analogous results were obtained for V46DA and Y331DA.

In the 3D structure of Fpn, two binding sites for cobalt have been identified: one formed by D39 and H43 and a second site which includes C326 and H507 [4,21]. V46 is close to the first cobalt binding site, and Y331 is relatively close to the second site, while Y54 and V161 are located in regions of the protein distant from the metal binding sites. Direct quenching of DA fluorescence by cobalt does not appear to be responsible for the observed changes, as titration of free DA with CoCl_2_ induces no substantial spectral variation (Figure S2). Thus, it can be assumed that conformational changes due to metal binding alter the polarity of the probe microenvironment, thus affecting emission λ_max_ and intensity. The λ_max_ blue shift indicates an increase in hydrophobicity. For V46DA, this also leads to an increase in fluorescence intensity, while for Y54DA, V161DA, and Y331DA a decrease is apparent. In the case of V46DA it is tempting to speculate that cobalt binding may induce a local conformational change that diminishes quenching by neighboring W42. It is known that tryptophan is able to quench fluorescence of (dimethylamino)naphthalene-based dyes [22].

Inspection of the recently reported structure of Fpn in occluded conformation (PDB 8C02, [20]) shows that the solvent accessible area is decreased mainly for Y331 (14 Å^2^), followed by Y54 (38 Å^2^), while V161 (41 Å^2^) and V46 (9 Å^2^) are only modestly affected. No experimentally determined 3D structure is available for human Fpn in the inward-open conformation, so the local environment of specific sidechains can only be inferred from the model, which suggests that V161 and V46 increase their solvent accessibility, at variance with Y54 and Y331.

Taken together, the observed spectral changes could be tentatively explained by a cobalt-induced transition from an outward-open to occluded conformation, consistent with structure-based solvent accessibility information and the local rearrangement of hydrogen bonding networks/van der Waals interactions. A more compact occluded conformation would lead to decreased polarity in the microenvironment for all four positions of the DA probe.

In conclusion, the genetic incorporation of DA in Fpn has been achieved and the potential of this approach for the analysis of conformational transitions induced by the binding of cobalt has been investigated. Future studies will address the effects of hepcidin and of Fe^2+^ to fully understand the details of the metal transport cycle of Fpn at the molecular level.

## 3. Materials and Methods

### 3.1. Vectors and Constructs

The pREAV vector carrying the tRNA/aaRS pair specific for dansylalanine (DA) was a generous gift from Prof. P.G. Schultz (The Scripps Research Institute). This vector allows expression of the DA-RS in *P. pastoris* under control of the methanol-inducible FLD1 promoter [18]. The plasmid was linearized with AatII prior to transformation in *P. pastoris* JC300 (*ade1, arg4, his4*) cells by electroporation.

The cDNA of wild-type human Fpn, Flag-tagged at the C-terminus, was cloned in pIB2 vector for constitutive expression under the control of the GAP promoter in the *P. pastoris* GS115 strain [7]. The pIB4 vector allows expression from the methanol-inducible AOX1 promoter [23] and it was used to construct the pADE vector by replacing the HIS4 auxotrophic marker with the *P. pastoris* ADE1 gene. Wild-type Flag-tagged Fpn was cloned in EcoRI/KpnI in pADE. Fpn mutants V46*amber*, Y54*amber*, V161*amber,* and Y331*amber* were obtained through site-directed mutagenesis using QuikChange II XL kit (Agilent, Santa Clara, CA, USA) or by overlap extension PCR. All constructs were sequence-verified by automated DNA sequencing at GATC Biotech/Microsynth (Gottingen, Germany). Plasmids were linearized with StuI (pIB2) or EcoNI (pADE) prior to transformation in *P. pastoris* JC300/DA-RS cells by electroporation.

Integration of all plasmids in the *P. pastoris* genome was confirmed by PCR on genomic DNA extracted from His+ (GS115) or Arg+, Ade+ (JC300) colonies.

### 3.2. Expression and Purification of Recombinant Fpn

For medium-scale expression of wild type Fpn, GS115 cells were grown in YPD (1 L) to OD_600_ 8–10 and harvested by centrifugation at 4500 rpm for 10 min. The cell pellet (10–15 mL) was resuspended in an equal volume of lysis buffer (MOPS 25 mM, NaCl 150 mM, pH 7.4, complete protease inhibitor cocktail (Roche, Monza, Italy), leupeptin and pepstatin 2 µg/mL, PMSF 1 mM, DNase 10 U/mL, RNase 2 µg/mL) and lysed by the glass beads method. Cell membranes were collected by ultracentrifugation at 35,000 rpm (Beckman 70Ti rotor) for 50 min and were washed with MOPS 25 mM, NaCl 300 mM, pH 7.4 and centrifuged at 35,000 rpm for 20 min. Membrane proteins were extracted in 15 mL extraction buffer (MOPS 25 mM, NaCl 150 mM, pH 7.4, Triton X-100 1%, leupeptin and pepstatin 2 µg/mL, PMSF 1 mM) for 1 h at room temperature, followed by centrifugation at 35,000 rpm for 20 min.

For medium-scale expression of dansylalanine (DA) Fpn *amber* mutants, yeast cells were grown in YPD (1 L) to OD_600_ 8–10, centrifuged, and induced in 200 mL BMMYH (yeast nitrogen base 0.67%, potassium phosphate buffer 50 mM pH 6.8, methanol 1%, histidine 50 µg/mL) supplemented with 1 mM DA (synthesized according to [17]) for 72–96 h adding 1% methanol daily. Cell lysis and membrane protein extraction were performed as described above for wild-type Fpn.

Fpn was purified by affinity chromatography on anti-Flag M2 agarose (Merck Sigma Aldrich, Milan, Italy) or anti-Flag G1 agarose (GenScript, Rijswijk, Netherlands). The resin (1 mL) was washed with 50–55 bed volumes of MOPS 25 mM, NaCl 300 mM, DDM 0.01% pH 7.0 to OD_280_ < 0.03 to remove contaminants and exchange Triton X-100 with dodecylmaltoside (DDM). The elution step was performed with MOPS/NaCl/DDM containing 0.1 mg/mL Flag peptide (3 mL) followed by glycine 100 mM, NaCl 150 mM, DDM 0.01% pH 3.5 (6 mL). Millipore Ultra 30 K devices were used to concentrate purified Fpn and exchange the protein with buffer when required. Protein content was measured with the microBCA assay (ThermoFisher, Monza, Italy). The monoclonal anti-human Fpn antibodies, 31A5 and 38G6, used for Western blot were a generous gift of Dr. Tara Arvedson (Amgen, Thousand Oaks, CA, USA).

### 3.3. Fluorescence Spectroscopy

Fluorescence spectra were obtained using a Jobin Yvon Fluoromax-3 spectrofluorometer. Protein samples were diluted in MOPS 25 mM, NaCl 150 mM, DDM 0.01% pH 7.0 and titrated with CoCl_2_ 2–60 µM. The excitation wavelength was 340 nm and the emission spectra were recorded between 400 and 650 nm at room temperature (slit width 5 nm for both excitation and emission). For cobalt titration of Fpn wild type (non-dansylated), excitation was at 295 nm and emission spectra were recorded between 300 and 450 nm. Titration data were fitted to a one-site binding model with GraphPad Prism 8. Cobalt was removed by gel-filtration on a PD10 prepacked column (GE-Cytiva, Milan, Italy) equilibrated in MOPS 25 mM, NaCl 150 mM, DDM 0.01% pH 7.0.

## Figures and Tables

**Table 1:** K_D_ values for cobalt.

Fpn	K_D_ (µM)
Wild type	7
V46DA	9
Y54DA	7
V161DA	14
Y331DA	6

## Data Availability

The data supporting results reported in this study are available in the current article.

## Supplementary Materials

The supporting information can be downloaded at: https://www.mdpi.com/article/10.3390/ijms241511919/s1.
