# Supplementary material for: Genetic Incorporation of Dansylalanine in Human Ferroportin to Probe the Alternating Access Mechanism of Iron Transport
Source: Int J Mol Sci. 2023 Jul 25;24(15):11919. doi: 10.3390/ijms241511919 (PMC10418311; doi:10.3390/ijms241511919)
Supplement: Supplementary file 1 [file ijms-24-11919-s001.zip › ijms-2493857-supplementary.pdf]

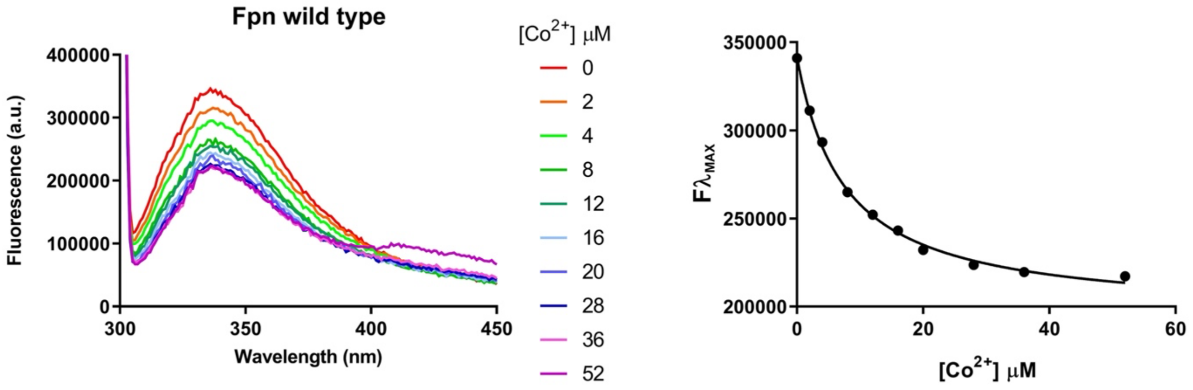

Figure S1. Fluorescence spectra of Fpn wild type titrated with cobalt. Left panel: emission spectra of Fpn 0.1 μM after addition of the indicated concentration of $CoCl_2$ were recorded in MOPS 25 mM pH 7.0, NaCl 150 mM, DDM 0.01%. Excitation was at 295 nm. Right panel: fluorescence changes at λ_{max}. Data were fit to a one-site binding model.

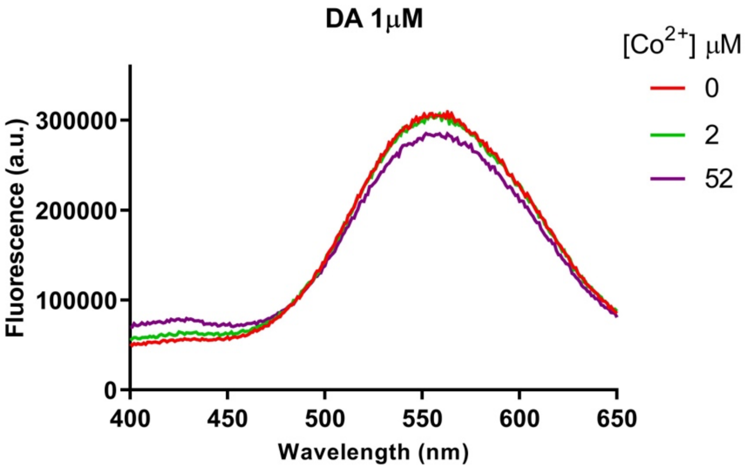

Figure S2. Fluorescence spectra of DA 1 μM titrated with cobalt. Emission spectra after addition of the indicated concentration of $CoCl_2$ were recorded in MOPS 25 mM pH 7.0, NaCl 150 mM, DDM 0.01%. Excitation was at 340 nm.
